# Increasing of Blood Brain Barrier Permeability and the Association With Depression and Anxiety in Systemic Lupus Erythematosus Patients

**DOI:** 10.3389/fmed.2022.852835

**Published:** 2022-03-29

**Authors:** Xiangyu Wang, Lihua Ma, Yuli Luo, Yifan Yang, Bibhuti Upreti, Yuqi Cheng, Ruomei Cui, Shuang Liu, Jian Xu

**Affiliations:** ^1^Department of Rheumatology and Immunology, First Affiliated Hospital of Kunming Medical University, Kunming, China; ^2^Department of Psychiatry, First Affiliated Hospital of Kunming Medical University, Kunming, China

**Keywords:** systemic lupus erythematosus, blood brain barrier, permeability, depression, anxiety

## Abstract

**Objective:**

To study changes in blood brain barrier (BBB) permeability in systemic lupus erythematosus (SLE) patients, and explore the association between the alterations in BBB permeability and depression/anxiety in SLE.

**Methods:**

Brain dynamic contrast enhanced magnetic resonance imaging (DCE-MRI) images were collected from 42 SLE patients and 23 healthy controls (HCs). Based on the Patlak pharmacokinetic model, the K^trans^ value of each voxel in the whole brain of each subject was calculated. BBB permeability indicator (the K^trans^ value) between SLE patients and healthy control group was compared. Hamilton Depression Scale (HAMD) and Hamilton Anxiety Scale (HAMA) were used to assess the mental health of SLE patients. The difference in BBB permeability was compared on SLE patients with depression/anxiety, SLE patients without depression/anxiety and HCs by ANOVA analysis.

**Results:**

The K^trans^ value of the right insular region of the SLE group was significantly higher than that of the healthy control group. And the K^trans^ value of the right insular region in SLE patients with depression/anxiety was significantly increased compared with SLE patients without depression/anxiety and HCs.

**Conclusions:**

SLE patients have increased BBB permeability, mainly in the right insular area. The increased BBB permeability in the right insular region is associated with the depression/anxiety in SLE patients.

## Introduction

Systemic lupus erythematosus (SLE) is an autoimmune disease, characterized by the production of multitude of autoantibodies affecting multiple systems, which leads to multiple organ damage. Neuropsychiatric systemic lupus erythematosus (NPSLE) is one of the most serious manifestations of SLE, affecting the peripheral and central nervous systems (CNS), which can occur at any time in the course of SLE. Depression and anxiety are the most common manifestations of diffuse CNS involvement in SLE (1). Genetic factors, immune response and blood brain barrier (BBB) dysfunction are all considered to be involved in the pathogenesis of NPSLE. Alexander et al. firstly found BBB damage in lupus mice (2). A clinical study also found that compared with healthy controls, SLE patients have increased BBB permeability, and patients with NPSLE have more severe BBB damage (3). In some diseases such as traumatic brain injury, epilepsy, and Alzheimer's disease, BBB functional damage has been proven to cause neuronal damage (4–6). Hence, we speculate that BBB damage caused by the influence of pathological changes underlying lupus makes abnormal BBB permeability, allowing inflammatory mediators in the peripheral circulation, such as cytokines, autoantibodies, to pass through the BBB and enter the CNS resulting in neuronal damage and a series of neuropsychiatric symptoms.

The traditionally accepted, reliable indicator for judging BBB damage is the albumin quotient (7). However, lumbar puncture is required to obtain the patient's cerebrospinal fluid (CSF), which is an invasive examination with related risks, so its clinical application is relatively limited. With the invention and development of medical imaging technology, a variety of imaging methods have been applied to the detection of BBB damage. This study used a non-invasive imaging technique that can quantitatively detect BBB dysfunction: dynamic contrast enhanced magnetic resonance imaging (DCE-MRI). The destruction of BBB can lead to the extravasation of low-molecular-weight magnetic resonance imaging contrast agents into the interstitial space outside blood vessels, resulting in increased signal at this site. DCE-MRI technology uses repeated brain scans to capture the changes in signal intensity caused by extravasation of intravenous low-molecular-weight contrast agents from the BBB, and uses the Patlak pharmacokinetic model to obtain the K^trans^ volume transfer constant, which can quantitatively reflect the destruction of BBB (8). The increase in the K^trans^ value represents an increase in the leakage of fluid through the BBB into the brain tissue. DCE-MRI technology has been used in the detection of BBB destruction in a variety of diseases, such as multiple sclerosis and brain tumors (9, 10).

Depression and anxiety as common emotional and mental disorders in SLE patients, present significantly higher incidence than which in the general population. Because of the lack of early symptoms and unified diagnostic criteria, depression and anxiety in SLE patients are often ignored in clinical practice. Increasing studies have shown that BBB dysfunction also plays an important role in the pathogenesis of depression. Animal experiments found that destruction of the BBB in mice produces depression-like behaviors (11). In addition, a recent study found that BBB destruction may be related to anxiety. Using DCE-MRI technology, Lyna et al. found that in patients with bipolar disorder, increased BBB permeability was associated with more severe depression and anxiety symptoms as well as the course of the disease (12).

At present, there are few studies that explored the association between BBB permeability changes and depression as well as anxiety in SLE patients. This study used DCE-MRI technology to detect changes in BBB permeability in SLE patients and evaluated depression and anxiety in SLE patients to further analyze their association with BBB dysfunction.

## Materials and Methods

### Participants

This study recruited patients with SLE who were admitted to the inpatient department (IPD) of the Department of Rheumatology and Immunology of the First Affiliated Hospital of Kunming Medical University from April 2016 to December 2017. All the patients fulfilled the 1997 revised American College of Rheumatology (ACR) classification and diagnostic criteria for SLE (13). The inclusion criteria include: (1) age ranging from 15 to 50 years old, (2) right-handedness, (3) participants willing to attend the study voluntarily and sign informed consents.

The exclusion criteria for all participants were as follows: (1) participants with a history of head trauma, (2) participants with a history of drug or alcohol dependence, (3) participants suffering from other connective tissue diseases, hematologic diseases, cardiovascular and cerebrovascular diseases, malignant tumors and renal insufficiency caused by non-SLE, (4) participants with parenchymal brain disease, CNS infection, epilepsy and other neuropsychiatric diseases not caused by SLE, etc., or with family history of neurological or psychiatric disease, (5) participants who have contraindications to MRI (such as pacemaker, metal implants in the body, history of contrast agent allergy, claustrophobia, glomerular filtration rate <30 ml/min, etc.), (6) participants currently pregnant or nursing. Structured Clinical Interview for DSM-IV Non-Patient Version (SCID-NP) for the healthy control is used to assess healthy participants. Using the Edinburgh Handedness Inventory to assess participants' handedness (14).

A total of 60 patients with SLE were initially enrolled. But 17 patients who could not complete all examinations were excluded, and one patient who had a clear history of depression was also excluded. Finally, 42 patients were included in the study. There were 23 healthy controls (HCs) whose age and sex matched the SLE group were recruited. An experienced rheumatologist and a psychiatrist performed the examinations of screening.

This research has been approved by the ethics committee of the First Affiliated Hospital of Kunming Medical University, Yunnan Province, China (ClinicalTrials.gov: NCT00703742). Before the start of the trial, each participant signed a written informed consent after being informed of the trial procedures in detail.

### Demographics and Psychological Assessment

We recorded the age, gender, weight, course of disease, previous history, family history, personal history of all enrolled patients and healthy volunteers. Then Hamilton Depression Scale (HAMD) and Hamilton Anxiety Scale (HAMA) were used to assess mental health of SLE patients. Scores on above mentioned scales were recorded and evaluated by two psychiatrists achieving good inter-examiner reliability after systematic training.

### DCE-MRI

All enrolled patients and healthy controls accepted a cranial T1 FFE sequence scan (incoherent gradient echo sequence spoiled GE) by the same operator under the same Philips 3.0T MRI scanner in the Imaging Department of the First Affiliated Hospital of Kunming Medical University on 3rd day after admission. Five whole-brain images under two flips of 2° and 12° were collected, and then the dynamic scan was performed when the flip angle was 12°. A total of 50 periods were scanned, and from the 4th period, the Gd-DTPA contrast agent was given in a rapid intravenous injection at a rate of 3 ml/s. The DCE-MRI parameters were set as follows:

T2W: TR = 2,500 ms, TE = 80 ms, fov = 235 × 208 mm, *t* = 45 s, matrix = 312 × 180, slices = 18, thickness = 6 mm, slice gap = 2 mm.

T1W: TR = 1,900 ms, TE = 20 ms, TI = 800 ms, fov = 230 × 190 mm, *t* = 1′14″, matrix = 232 × 139, slices = 18, thickness = 6 mm, slice gap = 2 mm.

DCE: TR = 8.2 ms, TE = 3.1 ms, fov = 220 × 220 mm, flip angles = 2°,12°, matrix = 148 × 128, slices = 40, thickness = 3 mm, slice gap = 0 mm.

### Image Processing

The original images were converted from the.dcm format to the.nii format via the dcm 2niigui software. For each subject, the first dynamic scan image was taken as the standard image, and all other images were registered to it. To correct the non-uniformity of B1 field, the 2° and 12° images were corrected by nu_correct in FSL software for N3 correction, and the mean value of the corrected uneven field was set to 1 (15). The drug concentration curve was obtained by measuring the relative value of signal enhancement E(t) at various concentrations of contrast agent C(t) (16). The K^trans^ was obtained based on the drug concentration curve and the Patlak model, and after processing, the K^trans^ image was finally produced (16).

### Statistical Analysis

IBM SPSS Statistics 21 was used for data statistical analysis. Quantitative data following normal distribution were compared by *t*-test and quantitative data with abnormal distribution was compared by Mann-Whitney *U*-test to evaluate the difference. Chi-square test was used to analyze binary variable. Ranked data were analyzed by non-parametric test. When *P* < 0.05, the difference was considered to be statistically significant.

We used DPABI software to perform an independent two sample *t*-test (Cluster size > 100, *P* ≤ 0.005 is statistically significant) to identify the area with the obvious difference of K^trans^ between SLE and HC groups. Then the three-dimensional image of that area was extracted to further calculate the K^trans^ value of each participant's whole brain and particular areas. ANOVA analysis was performed to compare the difference in the K^trans^ value of each voxel in the whole brain and particular regions between SLE patients with depression/anxiety, SLE patients without depression/anxiety, and HCs. The difference was considered to be statistically significant when the single voxel *P* ≤ 0.005 and the cluster volume > 100 voxels.

## Results

### Demographics of SLE and HC Groups

Totally 42 SLE patients and 23 HCs were included in this study. And there was no significant difference between the two groups on age and gender (Detailed data is shown in Table 1).

**Table 1:** Demographic of SLE and HC groups.

	**SLE (*n* = 42)**	**HCs (*n* = 23)**	***P***
Female (%)	33 (78.6)	18 (78.3)	0.977
Age (years) (IQR)	27.5 (23.0, 38.3)	27.0 (25.0, 33.0)	0.891

*SLE, systemic lupus erythematosus; HCs, healthy controls; IQR, interquartile range*.

### Clinical Data and Psychological Assessment of SLE Patients

Among 42 SLE patients, 52.4% of patients were suffering severe disease activity as well as 28.6% in moderate disease and 19% in mild disease. There were four patients who present symptoms of nervous system involvement. Scores of the psychological assessment scales showed that among 42 SLE patients, patients with depression accounted for 69.0% and patients with anxiety accounted for 47.6% of all patients. And all patients were accepting glucocorticoids treatment. Detailed data are shown in Table 2.

**Table 2:** Clinical features and psychological assessment of SLE patients.

	***N* (*n* = 42)**	**%**
**Disease activity (SLEDAI)**
Mild (≤ 6)	8	19.0
Moderate (7–12)	12	28.6
Severe (>12)	22	52.4
**Organ involvement**
Nervous system	4	9.5
Vasculitis	2	4.8
Articular and muscular	16	38.1
Renal	21	50.0
Cutaneous and mucous	21	50.0
Serositis	20	47.6
Hematological	17	40.5
**Autoantibodies (+)**
Anti-dsDNA antibody	20	47.6
Anti-Sm antibody	26	61.9
ARPA	14	33.3
Anti-nucleosome antibody	21	50.0
Anti-histone antibody	21	50.0
APLs (*n* = 41)^*^	12	29.3
**Depression/anxiety status**
Depression (HAMD ≥ 7)	29	69.0
Anxiety (HAMA ≥ 14)	20	47.6
**Treatment**
GCs	42	100.0
HCQ	36	85.7
CTX	28	66.7
MMF	7	16.7
CsA	4	9.5
MTX	5	11.9
LEF	7	16.7
Thalidomide	7	16.7
IVIG	5	11.9
Antiplatelet	11	26.2
Anticoagulation	22	52.4

*SLEDAI, systemic lupus erythematosus disease activity index; ARPA, Antiribosomal P protein antibodies; APLs, antiphospholipid antibodies; HAMD, Hamilton Depression Scale; HAMA, Hamilton Anxiety Scale; GCs, glucocorticoids; HCQ, hydroxychloroquine; CTX, cyclophosphamide; MMF, mycophenolate mofetil; CsA, cyclosporin A; MTX, methotrexate; LEF, leflunomide; IVIG, intravenous immunogloblin*.

* *APLs was available in 41 patients*.

### The Differences in BBB Permeability Between SLE and HC Groups

**Figure 1:**
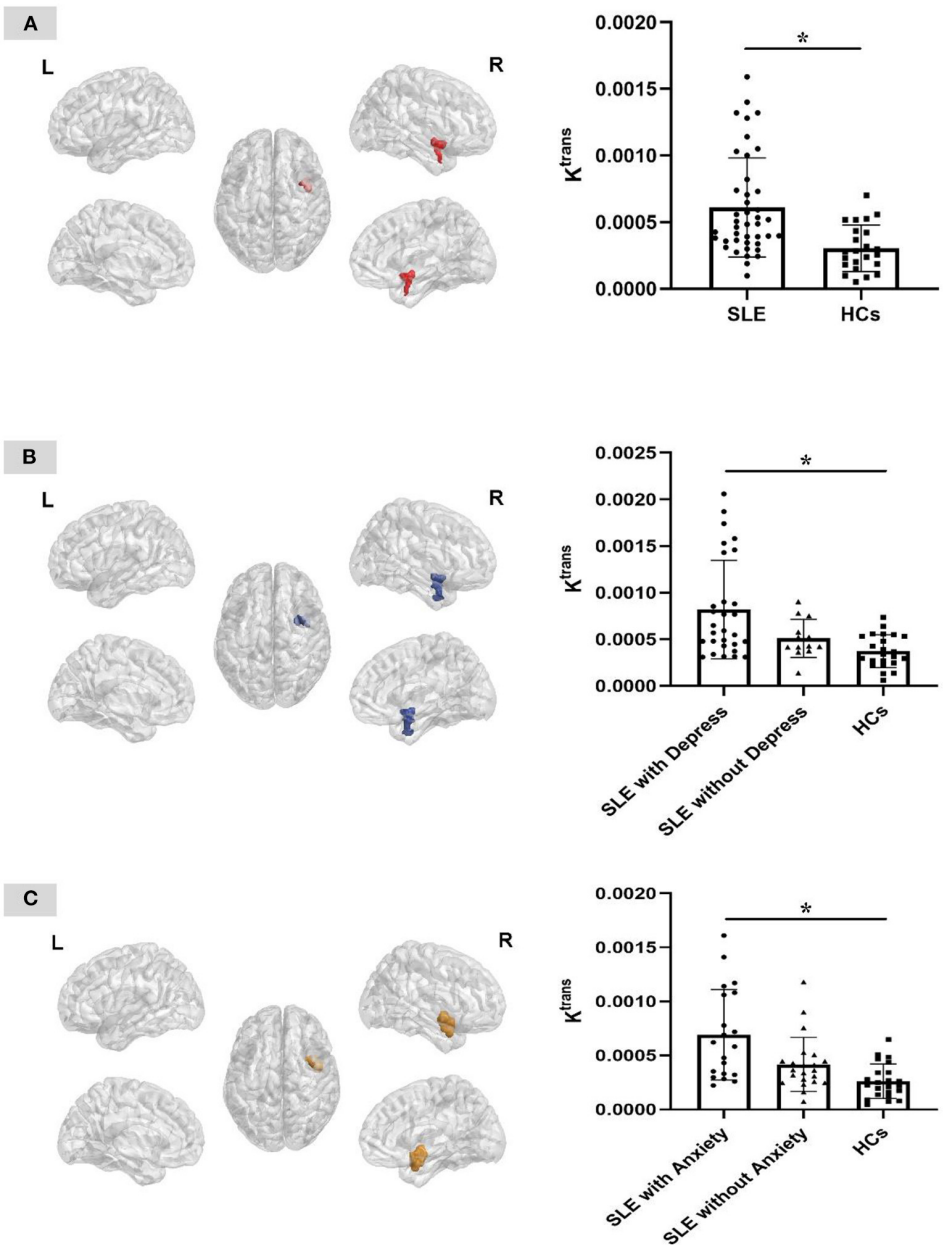
Three-dimensional plane view of the area with different K^trans^ value between groups. **(A)** The right insular region presents significantly higher K^trans^ value in the SLE group than that of the HC group. **(B)** The right insular region presents significantly higher K^trans^ value in the SLE patients with depression group than that of the patients without depression and the HC group. **(C)** The right insular region presents significantly higher K^trans^ value in the SLE patients with anxiety group than that of the patients without anxiety and the HC group. *P = 0.005, cluster size > 100. L, left; R, right.

We found the K^trans^ value significantly increased in the right insular area of the SLE group. So, we extracted the three-dimensional image of the right insular area, and calculated the average K^trans^ value of the whole brain and the K^trans^ value of the right insular area of the two groups, respectively. The K^trans^ value of the right insular region of the SLE group was significantly higher than that of the HC group (*P* < 0.005), while the average K^trans^ value of the whole brain was not statistically different between the two groups (Table 3; Figure 1A).

**Table 3:** The mean K^trans^ value of SLE and HC groups.

**Mean K^**trans**^ value**	**SLE (*n* = 42)**	**HCs (*n* = 23)**	***U***	***P***
Whole brain (×10^4)^	13.331 (11.251, 21.442)	13.161 (9.722, 18.709)	421	0.395
Right insula (×10^5)^	48.598 (35.378, 75.954)	28.017 (17.934, 43.300)	218	<0.001

*SLE, systemic lupus erythematosus; HCs, healthy controls; U, U-value from Mann-Whitney U-Test; P, P-value from Mann-Whitney U-Test*.

### The Difference in BBB Permeability Between SLE Patients With Depression/Anxiety, SLE Patients Without Depression/Anxiety and HCs

The results of ANOVA analysis showed that the K^trans^ value of the right insular region was significantly higher in SLE patients with depression than that of SLE patients without depression and HCs (*P* < 0.005), while there was no significant difference between SLE patients without depression and HCs. And the K^trans^ value of the right insular region was also significantly higher in SLE patients with anxiety than that of SLE patients without anxiety and HCs (*P* < 0.005), while there was no significant difference between SLE patients without anxiety and HCs (Table 4; Figures 1B,C).

**Table 4:** Regions with significantly different K^trans^ value in SLE patients and HCs.

**Cluster location**	**Peak (MNI)**			**Number of voxels**	***T*/F value**
	***x***	***y***	***z***		
**SLE patients and HCs**					
Right insula	42	6	−14	265	3.6876
**SLE patients with depression and HCs**					
Right insula	44	0	−12	436	3.446
**SLE patients with anxiety and HCs**					
Right insula	44	−2	−12	196	4.2160

*SLE, systemic lupus erythematosus; HCs, healthy controls*.

## Discussion

Compared with other invasive methods to evaluate the BBB dysfunction (17, 18), DCE-MRI not only exhibits good specificity and sensitivity but also can analyze the severity of BBB damage quantitatively. And as a non-invasive way, its' low risk will be easier to be accepted by patients.

In this study, the results showed that SLE patients have BBB dysfunction compared with HCs. And the increased BBB permeability was mainly located in the right insular area. In addition, to further explore whether the change of BBB permeability was associated with the emotional disorder of SLE patients, we divided patients into depression/anxiety groups and non-depression/non-anxiety groups to compare the degree of BBB damage in each subset. These results suggested that in SLE patients, the increased K^trans^ value in the right insula was associated with depression and anxiety.

A study using DCE-MRI technology to analyze the brain imaging of 6 SLE patients, found that the BBB permeability of SLE patients was increased compared with healthy controls (19), which was consistent with our results. And another study using DCE-MRI to assess BBB permeability found that BBB leakage was associated with gray matter loss and cognitive impairment (20), which were similar to the previous study using arterial spin labeling and diffusion-weighted brain MRI with a small sample size (21). Cagnoli et al. used magnetic resonance spectroscopy to study cell-level metabolic changes in several brain regions of SLE patients and HCs and found that compared with healthy controls and SLE patients without neuropsychiatric symptoms, neuron loss or damage was found in the right insular area of NPSLE patients (22). The abnormal brain area of NPSLE found in their study was consistent with the brain area of increased BBB permeability in SLE patients in our study.

Although there were several studies found that change of BBB permeability was associated with neuropsychiatric manifestations in SLE through other invasive methods (23–25), such as acute confusional state and cognitive dysfunction, our study was the first to explore the association between BBB permeability and depression/anxiety through the non-invasive method with relatively big sample size. This study not only provides evidence for extensive application of DCE-MRI to assess BBB damage in clinical management in SLE but also reveals that increased BBB permeability in the right insular region is associated with depression/anxiety in SLE patients.

Insula is a functional brain area involved in somatosensory and visceral sensation, regulating the conduction of pain and emotions, especially negative emotions (26). The anterior insula, as a component of ventral paralimbic regions, is an important part of the complex circuit initiating and regulating behavioral and emotional responses. In normal conditions, when the entire limbic-cortical depression network was activated, blood flow increased in the anterior insula (27). And in many neuropsychiatric diseases, abnormal damage of the insula and its network was found associated with emotional disorders. Frontotemporal dementia patients with damage of the anterior insula were found to present deficits in empathy and emotional reactivity (28). And the damage of insula networks was considered to contribute to depression in Parkinson's Disease (29). In major depression patients, bilateral amygdala and left anterior insula connectivity were found abnormally decreased in an affective network (30). These studies suggest that the destruction of structure in the insula and dysfunction of insular connectivity with other regions both can lead to depression.

Insula also plays an important role in anxiety (31). The severity of anxiety was considered to be positively correlated with central amygdala-insula functional connectivity (32), as well as somatic anxiety severity was found negatively correlated with the resting-state functional connectivity between anterior insula and medial prefrontal gyrus (33). The mechanism of the insula that takes part in anxiety is complex. In rats, the insula was found to have a direct role in anxiety with a regional difference. Medial and caudal regions of the insula exhibit an anxiolytic role, while the rostral region of the insula shows an anxiogenic role (34). Robinson et al. reveal another possible mechanism that anterior insula contributes to the maintenance of anxiety through consisting of a feedback loop with the prelimbic cortex to convey the interoceptive information from visceral change to the prelimbic cortex (35).

Furthermore, besides the direct role in depression/anxiety, the insula also takes part in the brain regulation of immunity. Neurons in the insular cortex can acquire and retrieve specific immune-related information and the insular cortex activity could induce or promote inflammation (36). Hence, we speculate that the high incidence of emotional disorder in SLE patients may not merely result from neuropsychiatric pathogenesis as conventionally known, but also may be associated with its immunity dysfunction and severity of inflammation affecting insula, and the BBB damage of the insular region may be a potential way. But further research is needed to explore the underlying mechanism.

## Limitations

There are also several limitations to our study. Firstly, the influence of various SLE treatment drugs could not be ruled out. For example, it has been reported that glucocorticoids have an impact on cognitive function (37). Recruiting untreated patients and comparing the DCE-MRI before and after treatment with a period of follow-up will be a reliable method to explore the effect of treatments on BBB damage. Secondly, the association of BBB damage with some clinical features could not be explored. Because of the limitation of sample size in this study, the number of patients in some subsets is insufficient to further analyze. Researches based on the larger sample size are needed.

In summary, this study found that SLE patients have increased BBB permeability, mainly in the right insular area. The increased BBB permeability in the right insular region is associated with depression/anxiety in SLE patients.

## Data Availability Statement

The original contributions presented in the study are included in the article/supplementary material, further inquiries can be directed to the corresponding authors.

## Ethics Statement

The studies involving human participants were reviewed and approved by Institutional Review Board of Kunming Medical University. Written informed consent to participate in this study was provided by the participants' legal guardian/next of kin.

## Author Contributions

XW, LM, and YL were responsible for the management of the research and drafting the article. YY was responsible for data analysis. BU was responsible for language editing. YC was responsible for the psychiatric assessment of participants. YC, RC, and SL provided invaluable research consultation. YC and JX oversaw the entire research project and the writing of the manuscript. All authors contributed to the article and approved the submitted version.

## Funding

This work was supported by grants from the National Natural Science Foundation of China (81760296, 82060259, and 81460256), Yunnan Province High-level health technical talents (leading talents) (L-2019004 and L-2019011), Yunnan Province Special Project for Famous Medical Talents of the Ten Thousand Talents Program (YNWR-MY-2018-040 and YNWR-MY-2018-041), the Funding of Yunnan Provincial Health Science and Technology Plan (2017NS051 and 2018NS0133), the Funding of Ministry of Science and Technology of Yunnan Province (2018ZF016), Yunnan Province Clinical Research Center for Skin Immune Diseases (2019ZF012), and Yunnan Province Clinical Center for Skin Immune Diseases (ZX2019-03-02).

## Conflict of Interest

The authors declare that the research was conducted in the absence of any commercial or financial relationships that could be construed as a potential conflict of interest.

## Publisher's Note

All claims expressed in this article are solely those of the authors and do not necessarily represent those of their affiliated organizations, or those of the publisher, the editors and the reviewers. Any product that may be evaluated in this article, or claim that may be made by its manufacturer, is not guaranteed or endorsed by the publisher.
